# An Innovative Immune Score-Based Prognostic Nomogram for Patients with Cervical Cancer

**DOI:** 10.1155/2020/8882576

**Published:** 2020-11-07

**Authors:** Xingling Qi, Yipeng Fu, Meng Zhang, Chong Lu, Yumeng Wang, Jing Peng, Guiling Li

**Affiliations:** ^1^Department of Integration of Western and Traditional Medicine, Obstetrics and Gynecology Hospital of Fudan University, Shanghai, China; ^2^Department of Breast Surgery, Obstetrics and Gynecology Hospital of Fudan University, Shanghai, China

## Abstract

**Background:**

In the past few years, the immune system and tumor immune microenvironment are becoming increasingly popular as more work has been accomplished in this field. However, nomograms based on immune-related characteristics for prognosis prediction of cervical cancer have not been fully explored to our knowledge. We constructed a novel immune score-based nomogram to predict patients with high risk and poor prognosis.

**Materials and Methods:**

198 patients with cervical cancer from The Cancer Genome Atlas (TCGA) database were included in our study. Immune scores were generated with Estimation of STromal and Immune cells in MAlignant Tumor tissues using Expression data (ESTIMATE) algorithm, and clinic-pathological characteristics were also included for subsequent analysis. Cox proportional hazards regression models were performed for univariate and multivariate analyses to screen the significant factors, and a prognostic nomogram was built. Bootstrap resampling analysis was used for internal validation. The calibration curve and concordance index (C-index) were used to assess the predictive performance of the nomogram.

**Results:**

Patients were split into three subgroups based on immune scores. We found that patients with high immune scores conferred significantly better overall survival (OS) compared with those with medium and low immune scores (hazard ratio (HR), 0.305; 95% confidence interval (CI), 0.108-0.869). A nomogram with a C-index of 0.720 had a favorable performance for predicting survival rate for clinical use by combining immune scores with other clinical features. The calibration curves at 3 and 5 years suggested a good consistency between the predicted OS and the actual OS probability.

**Conclusions:**

Our work highlights the potential clinical application significance of immune score-based nomogram in predicting the OS of cervical cancer patients.

## 1. Introduction

Cervical cancer is frequently lethal and is the most common female malignancy worldwide, representing 9.8% of all female cancers [1]. Cervical cancer is currently curable if detected and treated early. However, patients with advanced/recurrent cervical cancer carry a poor prognosis, which poses a severe threat to women health and life [2]. Given the high morbidity and poor survival rates related to cervical cancer, reliable prognostic tools are urgently needed to better identify populations at high risk and guide clinical treatments.

Numerous factors are associated with the prognosis of patients with cervical cancer, including tumor stage, depth of invasion, and lymph node (LN) status. The routine prognostic assessments of cervical cancer patients are currently based on the International Federation of Gynaecology and Obstetrics (FIGO) staging system [3]. However, this system is based on physical examination and cannot precisely predict the postoperative prognosis of patients. And clinical outcome differs even among patients with the same tumor stage [4]. Therefore, it is urgent to explore novel and efficient strategies to improve prognosis in patients with cervical cancer. Recently, accumulating evidence has demonstrated that the host immunological features are closely associated with tumor development and patients' prognosis [5–7].

Several studies have been performed on the association between tumor microenvironment (TME) and prognosis [8–11]. TME includes not only tumor cells, but also the surrounding immune cells and stromal cells. Tumor-infiltrating immune cells have been reported to play critical roles in the development of cervical cancer [12]. Besides, Yoshihara et al. calculated immune and stromal scores based on gene expression profiles and used these scores to infer the patterns of different infiltrating cells [13]. Furthermore, accumulating evidence has highlighted the potential clinical value of immune scores in the prognostic evaluation of patients with cervical cancer [14–16]. Therefore, a more practicable, effective predictive model using the immune scoring method compared to adopted staging systems should be introduced to assist clinicians to identify patients at higher risk of poor prognosis. And prognostic models based on immune scores have been implemented to predict survival rates in different tumor types [17–20]. However, as far as we know, nomograms based on immune scores for prognosis prediction of cervical cancer have not been well established.

In this study, we assessed immune scores of cervical cancer samples from TCGA database utilizing the ESTIMATE algorithm. We tried to discover the association between immune scores and the disease prognosis, and an immune score-based nomogram was constructed to predict the survival rate for individual patients.

## 2. Materials and Methods

### 2.1. Data Collection

The gene expression data of cervical cancer were obtained from TCGA database (https://cancergenome.nih.gov/). Clinic-pathological information and prognostic outcomes regarding TCGA cohort were retrieved from the cBioPortal website client (http://www.cbioportal.org), including unique patient IDs, age, race, tumor grade, tumor stage, clinical pathological type, survival status, and survival time [21]. Only subjects meeting the following inclusion criteria were included in this study: (a) pathologically diagnosed with cervical cancer, (b) with complete clinical pathological grading and staging information, and (c) with both follow-up information and expression data available at the same time. Samples with documented chemotherapy or targeted therapies were excluded to reduce possible confounding bias. And this study fully complies with TCGA publication guidelines and policies (https://cancergenome.nih.gov/publications/publicationguidelines).

### 2.2. Calculation of Immune Scores

ESTIMATE is a newly designed algorithm by Yoshihara et al. that can be used to calculate immune and stromal scores based on gene expression signatures [13]. These scores could reflect the patterns of different infiltrating cells in tumor samples. Specifically, by performing the single-sample gene set enrichment analysis (ssGSEA), gene expression values were then normalized and reordered. The enrichment score of each gene was calculated by the Empirical Cumulative Distribution Functions of the genes in the signature and the remaining genes. Immune and stromal scores were then obtained by comparing the difference between the cumulative distribution functions based on the absolute expression. Importantly, the ESTIMATE algorithm could be used extensively in almost all human solid cancers, including breast cancer, prostate carcinoma, colon cancer, and cervical cancer [14, 22–24]. Thus, this algorithm is a powerful method to evaluate the cellular heterogeneity of TME. In the present study, the immune scores were calculated and 95% CI was inferred from transcriptomic profiles of a cervical cancer cohort from TCGA database by ESTIMATE function of the R software package.

### 2.3. Data Preprocessing

**Figure 1:**
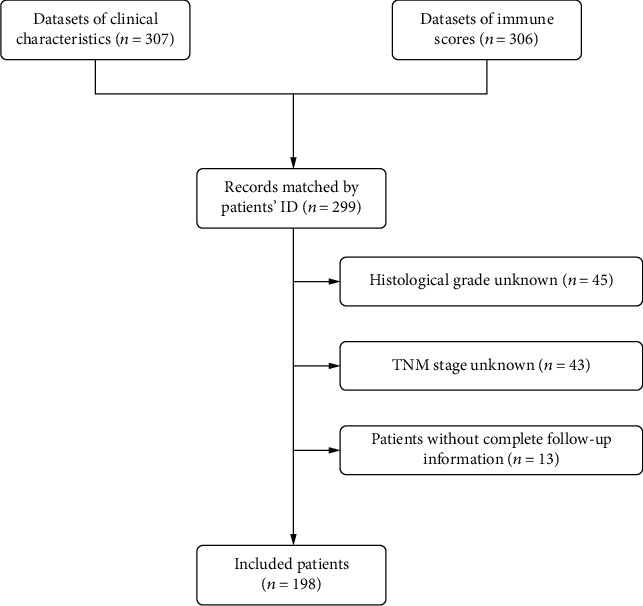
Flow diagram representing the main data processing procedure. TNM: tumor node metastasis.

Duplicate samples refer to those with the same serial sequencing number (sample ID) assigned. Sample data were firstly sorted by sample ID in an Excel spreadsheet (®Microsoft) to identify duplicates. Then, duplicate samples, as well as paracancerous samples, noncancerous samples, and samples without survival information were automatically removed using gdcRNA tools (version 4.6.3) [25]. We then matched the clinic-pathological variables and immune score information dataset based on sample IDs using the R “merge” function. As a result, a total of 198 cases were selected for further analysis. Each immune score corresponds to a different patient. The details of the cohort collections included and excluded at each stage of this study are listed in Figure 1.

### 2.4. Correlations between Immune Scores and Prognosis

OS was used as primary endpoint. It was defined as the time from inclusion to death from any cause. By applying the X-tile software (Yale University, version 3.6.1), the cutoff points of immune scores were determined [26]. Specifically, applying the X-tile plot in bioinformatics software, the best cutoff points of immune scores were determined according to the relationship between the immune score and OS. To assess significant differences between immune score subgroups and clinical pathological factors, the categorical variables were statistically analyzed using Pearson's chi-square test or Fisher's exact test, as appropriate (when any categorical data presented a value < 5 cases). And continuous variables were analyzed by the analysis of variance (ANOVA) for repeated measurements (one-way ANOVA or Kruskal-Wallis test). To further explore the relationship between immune score subgroups and prognosis, survival curves were generated using Kaplan-Meier estimates, and differences between immune score subgroups were assessed using the log-rank test.

### 2.5. Nomogram Construction and Validation

Single-factor logistic regression analysis was initially used to determine the relationship of baseline characteristics (Table 1) to the OS of patients. Then, to identify independent survival-related predictors, factors with statistical significance in single factor analysis were subjected to multifactor Cox regression analysis. After excluding the influence of stage and age, the adjusted hazard ratios for prognostic factors and the corresponding 95% CI were estimated. Subsequently, a nomogram model was established depending on the results of multivariate analysis. Internal validation was performed using 1000 bootstrap resamples to test the reliability of the nomogram. The predictive accuracy of the model was quantified by using the concordance index (C-index) [27]. The C-index uses values from 0.5 to 1, with 1.0 indicating perfect discriminative accuracy and 0.5 indicating lack of discrimination (no better than a coin flip). The calibration of the nomogram, which measures how far predictions are from observed outcomes, was assessed via calibration plots for 3- and 5-year survival rates of cervical cancer patients. All statistical significance tests were two-sided, and *P* < 0.05 was considered as the threshold of statistical significance. All descriptive statistics and tests were performed using the statistical software R version 3.6.0 (R Development Core Team 2011).

## 3. Results

### 3.1. Patients' Characteristics

**Figure 2:**
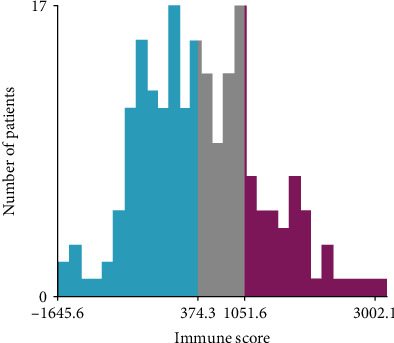
A histogram of the entire cohort divided into high, intermediate, and low immune score subgroups based on the cutoff values. The X-tile analysis was applied to determine the best cutoff value of immune scores. Blue represents the low immune score subgroup, gray represents the intermediate immune score subgroup, and red represents the high immune score subgroup.

After excluding no detailed data, a total of 198 study patients were enrolled in the final analysis. The detailed clinical characteristics were presented in Table 1. The patients ranged in age from 20 to 88 years (mean age, 48 years; SD, 13.77), and a significant proportion (35.86%) was older than age 50 years. Out of the 198 cervical cancer patients, 146 (77.66%) patients were Asian, 123 (65.43%) were in stage I, and 158 (84.04%) were cervical squamous cell carcinoma patients. The immune scores for 198 cervical cancer samples were downloaded from the ESTIMATE website. The immune scores of patients ranged from -1645.6 to 3002.1, with a median score of 302.42. Based on the best cutoff values generated by X-tile plots for immune scores (374.3 and 1051.6), patients were subsequently assigned to low, intermediate, and high immune score groups (X-tile plot was shown in Figure 2). In general, according to immune scores, patients were distributed as follows: 97 (48.9%) patients were in the low immune score subgroup, 60 (30.3%) patients were in the intermediate immune score subgroup, and 41 (20.7%) patients were in the high immune score subgroup. For all patients from the moment of initial diagnosis, the median OS time of the patients in this study was 33.68 months (range 0-210.51 months).

Table 1 showed the clinic-pathological characteristics of patient subgroups according to immune scores. In the low immune score subgroup, 69.1% of patients were less than 50 years of age. And in the high immune score subgroup, 56.1% of patients were less than 50 years of age. As for the disease stage, the results demonstrated that the percentage of those low immune score subgroup patients that were in stage IV was higher compared to the high score subgroup patients. Beyond this, most of the patients with high or intermediate immune scores were cervical squamous cell carcinoma cases.

### 3.2. Results of Univariate and Multivariate Analyses

**Figure 3:**
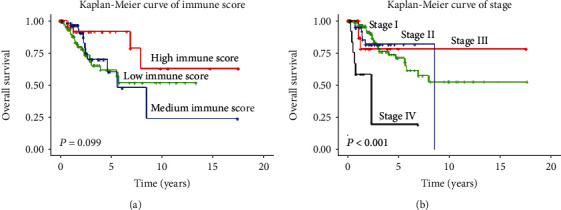
Kaplan-Meier survival curve delineating associations of (a) immune score subgroups and (b) stage subgroups with overall survival (OS) for patients with cervical cancer. In (a), green represents low immune scores, blue represents intermediate immune scores, and red represents high immune scores. In (b), green represents stage I, blue represents stage II, red represents stage III, and black represents stage IV.

The unadjusted and adjusted associations between clinical pathological features and OS rates were demonstrated in Table 2. As shown in Figure 3 and Table 2, there were substantial differences in terms of OS among patients with age of 70-80, high immune scores, and stage IV (unadjusted hazard ratio (HR) 3.680, 95% CI 1.145-11.828, and *P* = 0.029; HR 0.360, 95% CI 0.137-0.946, and *P* = 0.038; HR 6.944, 95% CI 2.924-16.488, and *P* = 1.12*e* − 05, respectively), while different races, grades, and pathological types indicated no statistical significance compared with OS.

All factors that displayed prognostic significance in the univariate analysis were included in the multivariate analysis.  Table 3 showed the results of multivariate logistic regression analysis on the determinants of OS. From the results, we can summarize that patients with high immune scores exhibited a better prognosis than those with low or intermediate immune scores (HR and 95% CI: 0.305 and 0.108-0.869, respectively). And, with one exception, we interestingly found that only the age of 70-80 years subgroup was strongly linked with a worse OS (HR: 5.722; 95% CI: 1.666-19.647). When compared to the patients in stages I, II, and III, patients in stage IV clearly had a worse OS (HRs and 95% CIs of stages II, III, and IV were 1.418 [0.584-3.445], 1.249 [0.357-4.364], and 5.898 [2.404-14.473], respectively). Furthermore, as for other clinical characteristics, significant associations were not found (*P* > 0.05).

### 3.3. Prognostic Nomogram for OS

**Figure 4:**
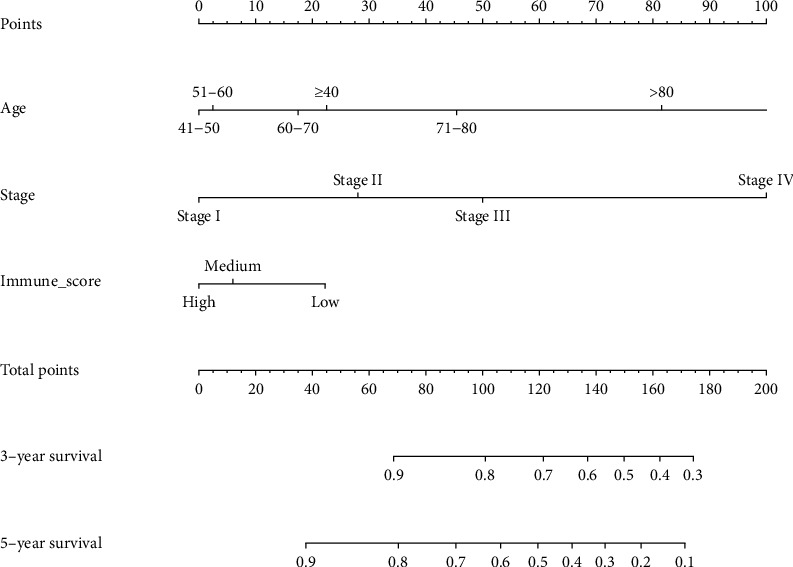
Nomogram for predicting 3- and 5-year overall survival (OS) probabilities in patients with cervical cancer. For utilizing the nomograms, an individual patient's value is plotted on each variable axis, and the line is drawn upwards to determine the number of points of each variable value was used. The sum of these numbers is located on the total points axis, and a line is drawn downward to the survival axes to determine the likelihood of 3- or 5-year survival.

**Figure 5:**
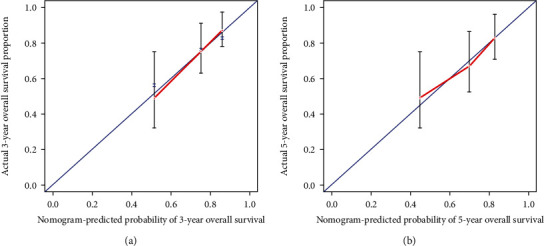
The calibration plot for nomogram predicted and observed (a) 3-year and (b) 5-year overall survival (OS) for cervical cancer. In calibration plots, nomogram-predicted OS is plotted on the *x*-axis; actual OS is plotted on the *y*-axis.

As shown in Figure 4, the prognostic nomogram combined all the important independent factors from multivariate analysis for the OS. A C-index of 1 indicates perfect prediction ability, and a C-index of 0.5 indicates a random guess. In the present study, the C-index of the established nomogram for predicting OS was 0.720 (95% CI, 0.622-0.818), which showed that the model had a good predictive ability. The calibration curve of 3-year and 5-year survival probabilities exhibited excellent concordance between actual observations and the nomogram predictions (Figures 5(a) and 5(b)).

## 4. Discussion

In this study, based on gene expression data and clinical information downloaded from the public databases, we were in a position to examine the prognostic risk factors in cervical cancer patients. The ESTIMATE algorithm has been confirmed to be an efficient method in large and independent datasets. According to this algorithm, the immune scores were obtained for each sample of cervical cancer from TCGA database. The samples were subsequently assigned to low, medium, and high immune score groups based on X-tile plots. Then, after consideration of potential confounders, our results revealed that high immune scores were surely associated with better OS of cervical cancer cases. Additionally, a nomogram combining immune scores with clinical factors was built up to efficiently predict the OS of cervical cancer patients, aiming at improving patients' prognosis.

The FIGO staging system is the most commonly used clinical staging system to estimate the prognosis for cervical cancer. However, this system has some limitations. Firstly, it is mainly based on the results of physical examinations and fails to incorporate other prognostic factors, including the host immune responses, pathological parameters, and lymph node metastasis [28]. Secondly, the prognostic outcome may significantly vary among patients with the same FIGO stage [29]. Since recently, there is growing evidence that immune-related characteristics could serve as prognostic indicators and guide a personalized treatment in the future. Several studies have identified a series of immune-related genes, which could serve as promising biomarkers for the prognostic prediction of cervical cancer [14, 16, 30]. These findings suggest that immune-related components play critical roles in the prognosis evaluation of patients with cervical cancer. However, most of these studies have not integrated clinic-pathological factors, and few have been widely popularized in routine clinical practice. Nomograms could integrate multiple clinical factors and give an individualized risk assessment for each patient. Compared with the traditionally used staging systems, nomograms have the obvious advantages and stronger predictive power [31].

The present study, to the best of our knowledge, is the first to combine the immune scores with clinical pathological characteristics to construct the nomogram for predicting the prognosis of cervical cancer patients. According to the results of our research, we found that after adjusting for potential confounding factors, high immune scores conferred apparently better OS than middle and low immune scores in patients with cervical cancer. One possible explanation could be that higher immune scores signified an increase in the extent of immune cell infiltration in the TME. And several previous studies have demonstrated that the immune microenvironment of cancer is an important prognostic factor [32–34]. Indeed, the TME induced and augmented the systemic antitumor immunity to effectively eradicate the tumor. In addition, novel immune-metabolic targets, such as GLUT1, which were used to overcome therapeutic resistance, played an independent prognostic value in cervical cancer [35]. And a study demonstrated that complete and durable regression of metastatic cervical cancer can occur after a single infusion of HPV-TILs [36]. Therefore, we speculate that immune score may not just be treated as a prognostic predictor, but also provide additional support for the investigation of immune-based treatments for this disease. To identify patients who were at high risk for poor prognosis is quite important because of the great health benefits that have been brought about by immunotherapy.

It should be noted that patients with higher immune scores seemed to be with cervical squamous cell carcinoma. It implied that patients with cervical squamous cell carcinoma may benefit from immunotherapy and achieve a better prognosis compared with those with cervical nonsquamous cell carcinoma, including endocervical adenocarcinoma and endocervical mucinous adenocarcinoma. Also, our results indicated that different pathological types were not related to the prognosis of cervical cancer. Actually, the roles of pathological types in cervical cancer so far remain controversial. Some studies in the literature found that histopathological types were of limited prognostic value or were changed merely in some selected variables or only within some subsets [37, 38]. A study based on the SEER data found that the survival differences between squamous cell carcinoma and adenocarcinoma did not exist [37]. And another study collected 17 histological subtype data of nonsquamous cell carcinoma of the uterine cervix from the Cancer Registry of Norway. This study found that histological subtypes lacked the statistical and clinical meaning except for small cell carcinoma, which was regarded as the only histologic subset of independent importance for prognosis [38]. Thus, our results were consistent with those of previous studies. However, a study conducted by Vinh-Hung et al. demonstrated that the histological type was an important independent prognostic factor in cervical cancer [10]. They found that small cell carcinoma and adenocarcinoma were associated with poorer survival. The inconsistent results of these studies may be due to diverse sample sources, different control groups, or by chance. All these results made this issue complicated and controversial. Further research is therefore needed to confirm our findings. Furthermore, for 70 to 80-year-old patients, their prognosis tended to be worse (HR: 5.722; 95% CI: 1.666-19.647), when compared to patients older than 80 years or younger than 70 years. The probable reason for this result may be due to the limited sample size in other age subsets.

We acknowledge that this study presents several limitations. First, it was a retrospective observational study based on publicly available databases, and it was difficult to cover the data from different races and geographical regions, so a prospective, multicenter, randomized clinical trial is needed to validate our findings. Second, many factors may affect the prognosis of cervical cancer patients, and additional research including more variables should be carried to improve the nomogram. Finally, because of the limited datasets including gene expression files available for calculating immune scores, our model needs to be further validated using independent data.

## 5. Conclusion

In summary, our research indicated that patients with high immune scores are significantly related to better OS. Moreover, we developed a novel nomogram based on immune scores and used it to calculate 3-year and 5-year survival rates, which may serve as a prognosis stratification tool for facilitating clinical decision-making to make a more reasonable follow-up plan.

## Figures and Tables

**Table 1:** Relationships between clinical pathological characteristics and immune scores in 198 cervical cancer patients.

		Immune scores			*χ* ^2^	*P* value
Characteristics	Total	≤374.3	374.3 to 1051.6	>1051.6		
Sample sizes	198	97 (48.9)	60 (30.3)	41 (20.7)		
Age (y)^a^					16.178	0.095
≤40	66	32 (33.0)	24 (40.0)	10 (24.4)		
40-50	61	35 (36.1)	13 (21.7)	13 (31.7)		
50-60	39	17 (17.5)	13 (21.7)	9 (22.0)		
60-70	22	11 (11.3)	8 (13.3)	3 (7.3)		
70-80	9	2 (2.1)	2 (3.3)	5 (12.2)		
>80	1	0 (0.0)	0 (0.0)	1 (2.4)		
Race					2.44	0.655
Asian	20	8 (8.2)	8 (13.3)	4 (9.8)		
White	146	76 (78.4)	41 (68.3)	29 (70.7)		
Others	32	13 (13.4)	11 (18.3)	8 (19.5)		
Grade					8.501	0.204
I	15	9 (9.3)	5 (8.3)	1 (2.4)		
II	96	52 (53.6)	26 (43.3)	18 (43.9)		
III	86	36 (37.1)	29 (48.3)	21 (51.2)		
IV	1	0 (0.0)	0 (0.0)	1 (2.4)		
Stage					4.748	0.577
I	123	61 (62.9)	38 (63.3)	24 (58.5)		
II	41	17 (17.5)	13 (21.7)	11 (26.8)		
III	22	10 (10.3)	7 (11.7)	5 (12.2)		
IV	12	9 (9.3)	2 (3.3)	1 (2.4)		
Histology					32.953	0.000
Cervical squamous cell carcinoma	158	62 (63.9)	56 (93.3)	40 (97.6)		
Cervical nonsquamous cell carcinoma	40	35 (36.1)	4 (6.7)	1 (2.4)		

^a^Age at diagnosis of cervical cancer.

**Table 2:** Univariate analyses of OS among cervical cancer patients based on clinicopathological features and immune scores.

		OS			
Characteristics	Total	Survival	Death	HR (95% CI)	*P* value
Age (y)^a^					
≤40	66	56 (84.8)	10 (15.1)	1.000	
40-50	61	46 (75.4)	15 (24.5)	1.511 (0.679, 3.365)	0.312
50-60	39	33 (84.6)	6 (15.3)	0.921 (0.334, 2.538)	0.874
60-70	22	16 (72.7)	6 (27.2)	1.551 (0.562, 4.278)	0.396
70-80	9	5 (55.5)	4 (44.4)	3.680 (1.145, 11.828)	0.029
>80	1	1 (100.0)	0 (0.0)	-	0.998
Immune scores					
≤374.3	97	73 (75.2)	24 (24.7)	1.000	
374.3 to 1051.6	60	48 (80.0)	12 (20.0)	0.813 (0.405, 1.632)	0.560
>1051.6	41	36 (87.8)	5 (12.1)	0.360 (0.137, 0.946)	0.038
Race					
Asian	20	18 (90.0)	2 (10.0)	1.000	
White	146	116 (79.4)	30 (20.5)	1.295 (0.308, 5.448)	0.725
Others	32	23 (71,8)	9 (28.1)	1.605 (0.343, 7.502)	0.548
Grade					
I	15	13 (86.6)	2 (13.3)	1.000	
II	96	71 (73.9)	25 (26.0)	1.611 (0.380, 6.826)	0.517
III	86	72 (83.7)	14 (16.2)	1.199 (0.271, 5.302)	0.811
IV	1	1 (100.0)	0 (0.0)	-	0.997
Stage					
I	123	99 (80.4)	24 (19.5)	1.000	
II	41	34 (82.9)	7 (17.0)	1.149 (0.491, 2.691)	0.748
III	22	19 (86.3)	3 (13.6)	1.204 (0.360, 4.023)	0.763
IV	12	5 (41.6)	7 (58.3)	6.944 (2.924, 16.488)	1.12*e*-05
Histology					
Cervical squamous cell carcinoma	158	124 (78.4)	34 (21.5)	1.000	
Cervical nonsquamous cell carcinoma	40	33 (82.5)	7 (17.5)	1.103 (0.401, 2.050)	0.813

Abbreviations: CI: confidence interval; ^a^Age at diagnosis of cervical cancer.

**Table 3:** Multivariate analyses of OS among cervical cancer patients based on clinicopathological features and immune scores.

Characteristics	OS	
	HR (95% CI)	*P* value
Age (y)^a^		
≤40	1.000	
40-50	1.285 (0.549,3.006)	0.563
50-60	0.931 (0.327, 2.653)	0.894
60-70	1.079 (0.375, 3.103)	0.888
70-80	5.722 (1.666, 19.647)	0.006
>80	-	0.998
Immune scores		
≤374.3	1.000	
374.3 to 1051.6	0.864 (0.412, 1.811)	0.699
>1051.6	0.305 (0.108, 0.869)	0.026
Stage		
I	1.000	
II	1.418 (0.584, 3.445)	0.441
III	1.249 (0.357, 4.364)	0.728
IV	5.898 (2.404, 14.473)	0.0001

Abbreviations: CI: confidence interval; ^a^Age at diagnosis of cervical cancer.

## Data Availability

All clinical pathological data of cervical cancer patients can be obtained from TCGA database and cBioPortal database. All immune score data can be downloaded from the ESTIMATE database (https://bioinformatics.mdanderson.org/estimate/).

## Supplementary Materials

Supplementary Materials Supplementary Table 1: the detailed clinical data for each patient. Supplementary Table 2: the immune scores of each patient based on the ESTIMATE algorithm. Supplementary Table 3: the main clinical characteristics and follow-up information of patients with cervical cancer. Supplementary Table 4: results of univariate Cox regression analyses. Supplementary Table 5: results of multivariate Cox regression analyses.

## References

[1] Bray F., Ferlay J., Soerjomataram I., Siegel R. L., Torre L. A., Jemal A. (2018). Global cancer statistics 2018: GLOBOCAN estimates of incidence and mortality worldwide for 36 cancers in 185 countries. *CA: A Cancer Journal for Clinicians*.

[2] Li H., Wu X., Cheng X. (2016). Advances in diagnosis and treatment of metastatic cervical cancer. *Journal of Gynecologic Oncology*.

[3] Pecorelli S., Benedet J. L., Creasman W. T., Shepherd J. H. (1999). FIGO staging of gynecologic cancer. *International Journal of Gynecology & Obstetrics*.

[4] Maeda K., Shibutani M., Otani H. (2015). Inflammation-based factors and prognosis in patients with colorectal cancer. *World Journal of Gastrointestinal Oncology*.

[5] Wolchok J. D., Chiarion-Sileni V., Gonzalez R. (2017). Overall survival with combined nivolumab and ipilimumab in advanced melanoma. *The New England Journal of Medicine*.

[6] Gandhi L., Rodríguez-Abreu D., Gadgeel S. (2018). Pembrolizumab plus chemotherapy in metastatic non-small-cell lung cancer. *The New England Journal of Medicine*.

[7] Koshkin V. S., Grivas P. (2018). Emerging role of immunotherapy in advanced urothelial carcinoma. *Current Oncology Reports*.

[8] Yang J., Li X., Liu X., Liu Y. (2015). The role of tumor-associated macrophages in breast carcinoma invasion and metastasis. *International Journal of Clinical and Experimental Pathology*.

[9] Lindsten T., Hedbrant A., Ramberg A. (2017). Effect of macrophages on breast cancer cell proliferation, and on expression of hormone receptors, uPAR and HER-2. *International Journal of Oncology*.

[10] Vinh-Hung V., Bourgain C., Vlastos G. (2007). Prognostic value of histopathology and trends in cervical cancer: a SEER population study. *BMC Cancer*.

[11] Hu K., Wang Z. M., Li J. N., Zhang S., Xiao Z. F., Tao Y. M. (2018). CLEC1B expression and PD-L1 expression predict clinical outcome in hepatocellular carcinoma with tumor hemorrhage. *Translational Oncology*.

[12] Wu Y., Ye S., Goswami S. (2020). Clinical significance of peripheral blood and tumor tissue lymphocyte subsets in cervical cancer patients. *BMC Cancer*.

[13] Yoshihara K., Shahmoradgoli M., Martínez E. (2013). Inferring tumour purity and stromal and immune cell admixture from expression data. *Nature Communications*.

[14] Pan X. B., Lu Y., Huang J. L., Long Y., Yao D. S. (2019). Prognostic genes in the tumor microenvironment in cervical squamous cell carcinoma. *Aging (Albany NY)*.

[15] Liu J., Wu Z., Wang Y. (2020). A prognostic signature based on immune-related genes for cervical squamous cell carcinoma and endocervical adenocarcinoma. *International Immunopharmacology*.

[16] Ma J., Cheng P., Chen X., Zhou C., Zheng W. (2020). Mining of prognosis-related genes in cervical squamous cell carcinoma immune microenvironment. *PeerJ*.

[17] Shen Q., Hu G., Wu J., Lv L. (2020). A new clinical prognostic nomogram for liver cancer based on immune score. *PLoS One*.

[18] Huang S.-N., Li G.-S., Zhou X.-G. (2020). Identification of an immune score-based gene panel with prognostic power for oral squamous cell carcinoma. *Medical Science Monitor*.

[19] Wang J., Zhang C., Li A. (2020). A prognostic nomogram based on immune scores predicts postoperative survival for patients with hepatocellular carcinoma. *BioMed Research International*.

[20] Jiang S., Zhang J., Bian J. (2020). Novel nomograms based on immune and stromal scores for predicting the disease-free and overall survival of patients with hepatocellular carcinoma undergoing radical surgery. *Journal of Surgical Oncology*.

[21] Gao J., Aksoy B. A., Dogrusoz U. (2013). Integrative analysis of complex cancer genomics and clinical profiles using the cBioPortal. *Science Signaling*.

[22] Shah N., Wang P., Wongvipat J. (2017). Regulation of the glucocorticoid receptor via a BET-dependent enhancer drives antiandrogen resistance in prostate cancer. *eLife*.

[23] Priedigkeit N., Watters R. J., Lucas P. C. (2017). Exome-capture RNA sequencing of decade-old breast cancers and matched decalcified bone metastases. *JCI Insight*.

[24] Alonso M. H., Aussó S., Lopez-Doriga A. (2017). Comprehensive analysis of copy number aberrations in microsatellite stable colon cancer in view of stromal component. *British Journal of Cancer*.

[25] Li R., Qu H., Wang S. (2018). GDCRNATools: an R/bioconductor package for integrative analysis of lncRNA, miRNA and mRNA data in GDC. *Bioinformatics*.

[26] Camp R. L., Dolled-Filhart M., Rimm D. L. (2004). X-tile: a new bio-informatics tool for biomarker assessment and outcome-based cut-point optimization. *Clinical Cancer Research*.

[27] Kang L., Chen W., Petrick N. A., Gallas B. D. (2015). Comparing two correlated C indices with right-censored survival outcome: a one-shot nonparametric approach. *Statistics in Medicine*.

[28] Kupets R., Covens A. (2001). Is the International Federation of Gynecology and Obstetrics staging system for cervical carcinoma able to predict survival in patients with cervical carcinoma? An assessment of clinimetric properties. *Cancer*.

[29] Kyung M. S., Kim H. B., Seoung J. Y. (2015). Tumor size and lymph node status determined by imaging are reliable factors for predicting advanced cervical cancer prognosis. *Oncology Letters*.

[30] Nie H., Bu F., Xu J., Li T., Huang J. (2020). 29 immune-related genes pairs signature predict the prognosis of cervical cancer patients. *Scientific Reports*.

[31] Wang Y., Li J., Xia Y. (2013). Prognostic nomogram for intrahepatic cholangiocarcinoma after partial hepatectomy. *Journal of Clinical Oncology*.

[32] Qian B. Z., Pollard J. W. (2010). Macrophage diversity enhances tumor progression and metastasis. *Cell*.

[33] Robinson B. D., Sica G. L., Liu Y. F. (2009). Tumor microenvironment of metastasis in human breast carcinoma: a potential prognostic marker linked to hematogenous dissemination. *Clinical Cancer Research*.

[34] Klemm F., Joyce J. A. (2015). Microenvironmental regulation of therapeutic response in cancer. *Trends in Cell Biology*.

[35] Kim B. H., Chang J. H. (2019). Differential effect of GLUT1 overexpression on survival and tumor immune microenvironment of human papilloma virus type 16-positive and -negative cervical cancer. *Scientific Reports*.

[36] Stevanović S., Draper L. M., Langhan M. M. (2015). Complete regression of metastatic cervical cancer after treatment with human papillomavirus-targeted tumor-infiltrating T cells. *Journal of Clinical Oncology*.

[37] Kosary C. L. (1994). FIGO stage, histology, histologic grade, age and race as prognostic factors in determining survival for cancers of the female gynecological system: an analysis of 1973-87 SEER cases of cancers of the endometrium, cervix, ovary, vulva, and vagina. *Seminars in Surgical Oncology*.

[38] Alfsen G. C., Kristensen G. B., Skovlund E., Pettersen E. O., Abeler V. M. (2001). Histologic subtype has minor importance for overall survival in patients with adenocarcinoma of the uterine cervix: a population-based study of prognostic factors in 505 patients with nonsquamous cell carcinomas of the cervix. *Cancer*.

